# Occurrence of *Trichinella* spp. in wild animals in northwestern Libya

**Published:** 2013-07-22

**Authors:** M.M. Hosni, A.A. El Maghrbi, K.S. Ganghish

**Affiliations:** 1 *Department of Preventive Medicine, Faculty of Veterinary Medicine, University of Tripoli, P. O. Box 13662, Tripoli, Libya*; 2 *Department of Microbiology and Parasitology, Faculty of Veterinary Medicine, University of Tripoli, P. O. Box 13662, Tripoli, Libya*

**Keywords:** Libya, *Trichinella* spp, Wild animals, Zoonosis

## Abstract

The present study determined the occurrence of *Trichinella* spp. in captured and some perished wildlife animals which included 70 hedgehogs, 19 red foxes, 13 common jackals and 8 crested porcupines in northwestern Libya. Muscle samples of these animals were examined by trichinoscopy. *Trichinella* larvae were detected only in 4 (5.7%) of the hedgehogs (*Erinaceus algirus*) and 2 (10.5%) of the red foxes (*Vulpes vulpes*). Larvae were found in the muscles of the diaphragm, abdomen, tongue, forelimb, hindlimb and intercostal muscles. Examination of tissue sections revealed the presence of numerous cysts within the muscle fibers containing one or more coiled or elongated larvae. Inflammatory cell infiltration was observed around the cysts especially at their poles. Results indicated the importance of wild animals as reservoirs of *Trichinella* larvae and their role in the transmission of the disease to other wild and domestic animals as well as humans.

## Introduction

Parasitic diseases in wild animals are of great importance in both human and veterinary medicine. Wildlife is now recognized as an important source of emerging human pathogens, including parasites (Polley, 2005). These diseases may be caused by harmful parasites such as *Toxoplasma gondii* and *Trichinella spiralis*, in which the wild animals act as a reservoir and a carrier for such parasites (Krastad and Nestel, 1981).

Out of 198 countries, *Trichinella* spp. infection has been documented in domestic animals and in wildlifein43 (21.9%) and 66 (33.3%) countries, respectively (Pozio, 2007).

Ecological imbalance in sub-Sahara of Libya due to desertification, shortage of food and water during last 25 years led to immigration of wild animals from their natural geographical zone to nearby inhabited areas of man and domestic animals, accordingly, wild animals such as foxes, hedgehogs, common jackals, porcupines, hares and jerboa are observed to live side by side with domestic animals and humans. Such contact allows the transmission of disease agents from wild animals to domestic animals and humans.

The life cycle began when meat containing viable infective *Trichinella* larvae is eaten. The cysts are freed from the tissues in the stomach and the larvae encyst there or in the duodenum and jejunum. Then, they invade the mucosal epithelium and rapidly developed through four larval stages, each of which is terminated by a molt (Kozek, 1971a, b).

The data concerning the species, population and geographical distribution of wild animals in Libya are scarce. Consequently, the diseases that occur in these animals are also not well studied and documented.

Few reports are available regarding the prevalence of trichinosis in wildlife animals. Hence, this study was undertaken to determine the prevalence of trichinosis in free range foxes, hedgehogs, common jackals and crested porcupines in the northwestern part of Libya.

## Materials and Methods

### Study area

**Fig. 1:**
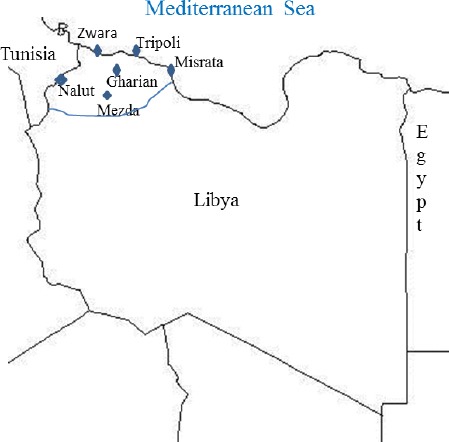
Geographical map showed the studied area (northwestern part of Libya).

This study was conducted in the northwestern part of Libya (Fig. 1). This area is considered to be a grazing area for Libya’s livestock which includes more than 50% of the five million sheep and goats, less than 20% of 102,509 cattle and about 109,397 of camels (LGIA, 2007). It is also inhabited by several species of wild animals of which some were considered as endangered species (FAO, 1992).

### Collection of samples

One hundred and ten different wildlife hosts belonging to three families were captured from several localities during the period of June 2008 till December 2009. From family Canidae: 19 red foxes, 13 common jackals, and from family Hystricidae: 8 crested porcupines were captured alive in their natural habitat using special nets, steel leg hold traps and steel cages. Some perished animals were collected from road sides. From family Erinaceidae: 70 hedgehogs were collected alive from farms and road sides at night by hand using protective gloves.

The identification of captured animals was done according to Dorst and Dondelot (1990). Carcasses were preserved in 10% formalin in special jars and transported immediately to the laboratory.

### Muscles examination

The euthanized and perished animals were dissected, skinned, eviscerated and examined carefully for internal parasites. Ten grams of abdominal, hindlimb, forelimb and diaphragm muscles (whole diaphragm of hedgehog) were examined by using a small unstained squashed preparation of muscle which was compressed by trichinoscopy. The preparation was then examined under the light microscope. This method allowed the examination of relatively more tissue than that by using histological examination.

### Histopathology examination

Tissue samples were taken from the infected muscles of infected animal. All tissue specimens were fixed in 10% neutral formalin. The fixed tissues were dehydrated in ascending grades of alcohol, cleared in xylol, embedded in paraffin (Lillie and Fullmer, 1976), then sectioned at 4 micron, and lastly stained according to Carleton *et al*. (1967).

## Results

From the 70 examined hedgehogs only 4 (5.7%) and 2 (10.5%) of the 19 red foxes were found infected with *Trichinella* spp. None of the common jackals and crested porcupines was found infected (Table 1). The abdomen, forelimb and hindlimb muscles were the most infected.

**Table 1:** Prevalence rate of *Trichinella* spp. in different wildlife hosts.

Animal	No. of animals examined	No. of animals infected	%
Hedgehog (*Erinaceus algirus*)	70	4	5.7
Red fox (*Vulpes vuples*)	19	2	10.5
Common jackal (*Canis aureus*)	13	0	0
Crested porcupine (*Hystrix cristata*)	8	0	0

### Histopathological results

**Fig. 2:**
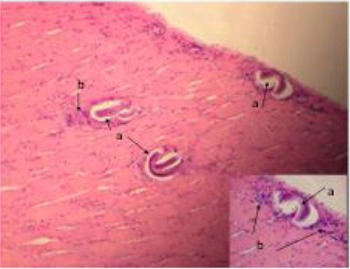
(a) Muscle section showing coiled larval encysted between muscle bundles. (b) Inflammatory cells infiltration was observed around most of the cysts especially at their poles.

The tissue sections revealed the presence of many cysts within the muscle fibers. The cysts were mostly lemon shape, some were elongated, and few oval and round cysts contained one or more coiled or elongated larvae. The small cysts were rounded by homogenous pale eosinophilic capsules, whereas most cysts were surrounded by a thin capsule. The muscle fibers were swollen and lost their cross striations. Mononuclear inflammatory cell infiltrations and a few numbers of eosinophilic leukocytes were observed around most of the cysts, especially at their poles. Focal aggregations of mononuclear cells were observed between some muscle fibers and bundles (Fig. 2).

## Discussion

In the present study the prevalence of *Trichinella* spp. was 5.7% and 10.5% in hedgehogs and red foxes, respectively. This prevalence in red foxes was lower than that 8.5% recorded by Prestrud *et al*. (1993) in Svalbard and in Spain 8.9 % reported by Criado-Fornelio *et al*. (2000).

On the other hand, the prevalence of the *Trichinella* spp. in this study was higher than that obtained by Enemark *et al*. (2000) in Denmark, Smith *et al*. (2003) in England, Zimmer *et al*. (2009) in Ireland, La Rosa *et al*. (1991) in France and Appleyard *et al*. (1998) in Canada who recorded a lower prevalence rate of 0.001%, 0.6 %, 0.2%, 0.96 and 1.7% respectively. Nearly, similar prevalence was reported in Poland 4.4% (Balicka-Ramisz *et al.*, 2007). However, Ballek *et al*. (1992) in Germany and Magi *et al*. (2009) in Italy did not find *T. spirals* in 397 and 129 red foxes examined respectively.

Trichinosis is one of the most important zoonotic problem world-wide. Pedro and Boris (1989) stated that *T. spiralis* has a wide range of hosts among domestic and wild animals. The infection has been confirmed in 104 species of mammals; 58 species of carnivores, 27 rodents, 7 insectivores and 12 species in other orders. The present study detected the *Trichinella* spp. in two species of mammals, one of them is carnivores (red foxes) and the other one is insectivores (hedgehog). These results are similar to that recorded for the first time by Hosni, (2006) were *Trichinella* was found in red fox and hedgehog with prevalence rate of 7.7% in both while it was recorded in the two other investigated mammal species; common Jackal (*Canis aureus*) and crested porcupine (*Hystrix cristata*).

Holliman and Meade (1980) reported that the main reservoir of *T. spiralis* in the nature is wild carnivores. The fox is an important reservoir in Europe because of its population density and high infection rates. While Nelson *et al*. (1963) reported that in Sub-Sahara Africa, only a wild cycle is known and the parasite is widely distributed among wild carnivores. *Trichinella britovi*, *Trichinella nelsoni* and *Trichinella zimbabwensis* and one genotype (Trichinella T8) are known to occur in sub-Saharan Africa. Distinct geographic ranges with overlapping of some taxa in some areas have been observed. Genetic variants of *T. nelsoni* has been reported to occur among parasites originating from Eastern and Southern Africa and sequence heterogeneity also occurs among *T. zimbabwensis* isolates originating from different regions of Zimbabwe and South Africa. Field observations so far indicate that sylvatic *Trichinella* infections in the region are common in carnivores (mammals and reptiles) and to a lesser extent in omnivores (Mukaratirwa *et al.*, 2013). In countries of northern Africa bordering the Mediterranean, human cases with trichinosis were reported in Algeria and Egypt (Pozio, 2007).

Trichinosis in this study was detected in two species of captured animals; the red fox and hedgehog. These free-ranging wild animals may play an important role as wildlife reservoir in the urban and rural areas where human activities take place. The zoonoses with a wildlife reservoir represent a major public health problem, affecting all continents. The importance and recognition of wildlife as a reservoir of zoonoses are increasing (Kruse *et al.*, 2004). Our results may form the basis for evaluating the risk of parasite transmission to the domestic cycle and human beings. Further studies are required for the molecular characterization of *Trichinella* larvae isolated.
